# A study to evaluate association of nuclear grooving in benign thyroid lesions with *RET/*PTC1 and *RET/*PTC3 gene translocation

**DOI:** 10.1186/s13044-023-00161-9

**Published:** 2023-07-03

**Authors:** Basavaraj Rangalakshmi Ashwini, Chandran Nirmala, Muthuvelu Natarajan, Dayananda S Biligi

**Affiliations:** Department of Pathology, Bangalore Medical College and Research Institute, Bengaluru, Karnataka India

**Keywords:** Benign thyroid lesions, Nuclear grooving, RET/PTC1, And RET/PTC3 gene translocation

## Abstract

**Introduction:**

Papillary thyroid carcinoma (PTC) is the most common malignant lesion of the thyroid characterized by unique histological features like nuclear grooving, nuclear clearing, and intra-nuclear inclusions. However, nuclear grooves are observed even in benign thyroid lesions (BTL) like nodular goiter (NG), Hashimoto's thyroiditis (HT), and follicular adenoma (FA) resulting in diagnostic dilemma of the presence of PTC in such BTL. RET/PTC gene translocation is one of the most common oncogenic rearrangements seen in PTC, known to be associated with nuclear grooving. Among different types of RET/PTC translocations, RET/PTC1 and RET/PTC3 gene translocations are the most common types. These translocations have also been identified in many BTL like hyperplastic nodules and HT. Our study aimed to determine the frequency of nuclear grooving in BTL and evaluate their association with RET/PTC1 and RET/PTC3 gene translocation.

**Methods:**

Formalin-fixed, paraffin-embedded (FFPE) tissue blocks of NG, HT, and FA were included in the study. The hematoxylin and eosin (H&E) stained sections were evaluated for the presence of nuclear grooving/high power field (hpf) and a scoring of 0 to 3 was used for the number of grooves. Sections of 10 μ thickness were cut and the cells containing the nuclear grooves were picked using Laser-Capture microdissection. About 20 to 50 such cells were microdissected in each of the cases followed by RNA extraction, cDNA conversion, realtime-polymerase chain reaction (RQ-PCR) for RET/PTC1 and RET/PTC3 gene translocation, and the findings were analyzed for statistical significance.

**Results:**

Out of 87 BTL included in the study, 67 (77.0%) were NG, 12 (13.7%) were HT, and 8 (9.2%) were FA. Thirty-two cases (36.8%) had nuclear grooving with 18 out of 67 NG, 6 out of 12 HT, and all 8 cases of FA showing a varying number of nuclear grooves. A significant association between the number of nuclear grooves with RET/PTC gene translocation (*p*-value of 0.001) was obtained. A significant association of HT with RET/PTC gene translocation (*p*-value of 0.038) was observed. RET/PTC1 and RET/PTC3 translocation were seen in 5 out of 87 cases, with HT showing positivity in 2 and FA in 1 case for RET/PTC1 and HT in 1 and FA in 2 cases for RET/PTC3 gene translocation with 1 case of FA being positive for both RET/PTC1 and RET/PTC3 gene translocation.

**Conclusions:**

The frequency of nuclear grooving among BTLs in our study was 36.8%. Our study shows, that when BTLs, show nuclear grooves, with an increase in the nuclear size, oval and elongated shape, favors the possibility of an underlying genetic aberration like RET/PTC gene translocation, which in turn supports the reporting pathologist to suggest a close follow up of the patients on seeing such nuclear features on cytology or histopathology sample, particularly in HT.

## Introduction

Papillary carcinoma of the thyroid (PTC) is one of the most common malignant lesions of the thyroid characterized by unique histological features like papillary pattern, nuclear grooving, nuclear clearing, and intra-nuclear inclusions [1, 2]. Nuclear grooving which is an important diagnostic criterion in PTC is sometimes observed even in benign conditions like nodular goiter (NG), Hashimoto’s thyroiditis (HT), and follicular adenoma (FA) resulting in the diagnostic dilemma of the presence of PTC in such benign thyroid lesions (BTL) [3]. RET/PTC gene rearrangement is one of the common genetic alterations seen in PTC, causing oncogenic rearrangement of RET gene and accounting for about 20–40% of adult sporadic PTC [4, 5]. RET/PTC rearrangement occurs as a genetic event following recombination between the 3′ tyrosine kinase portion of RET and the 5′ portion of a partner gene. Depending upon the type of the partner gene, 13 different types of RET/PTC translocations have been described. The CCDC6, also known as the H4 gene, is a 5’ partner gene with RET/PTC 1, and NCOA4, also known as ELE1 acts as a 5’ partner gene to the 3' tyrosine domain of RET gene in RET/PTC3, which are the most common type of RET/PTC translocations [6]. In recent times, these translocations have also been identified in many benign thyroid lesions (BTL) like hyperplastic nodules and Hashimoto's thyroiditis (HT) [7–10]. It has been shown that benign thyroid lesions with RET/PTC translocation have a rapid growth rate necessitating surgery [11]. RET/PTC gene translocation is known to be associated with nuclear irregularity in thyrocytes, including nuclear grooving and intranuclear inclusions [12].

We intended to determine the frequency of nuclear grooving in BTLs and evaluate their association with RET/PTC1 and RET/PTC3 gene translocation with the research question of whether the BTL showing nuclear grooving has RET/PTC gene translocations resulting in such nuclear abnormality and whether nuclear grooving can be a surrogate marker for RET/PTC gene translocation in BTL necessitating further follow-up of the patients.

## Materials and methods

### Sample collection

The study was conducted after obtaining ethical committee clearance from the institution. It was a retrospective study conducted between January 2018 to December 2019. It included 87 randomly selected thyroid specimens, which were histopathologically diagnosed as BTLs in the department of Pathology out of 145 cases of BTLs, diagnosed during the study period. The patients were subjected to thyroid surgeries with a clinical history of thyroid enlargement which included; diffuse thyroid enlargement in 14 cases (10%), solitary thyroid nodule in 26 cases (29.8%), and multinodular goiter in 47 cases (54%). The formalin-fixed, paraffin-embedded (FFPE) blocks of eighty-seven histopathologically confirmed BTL were retrospectively retrieved and included in the study.

### Histopathological examination

**Fig. 1:**
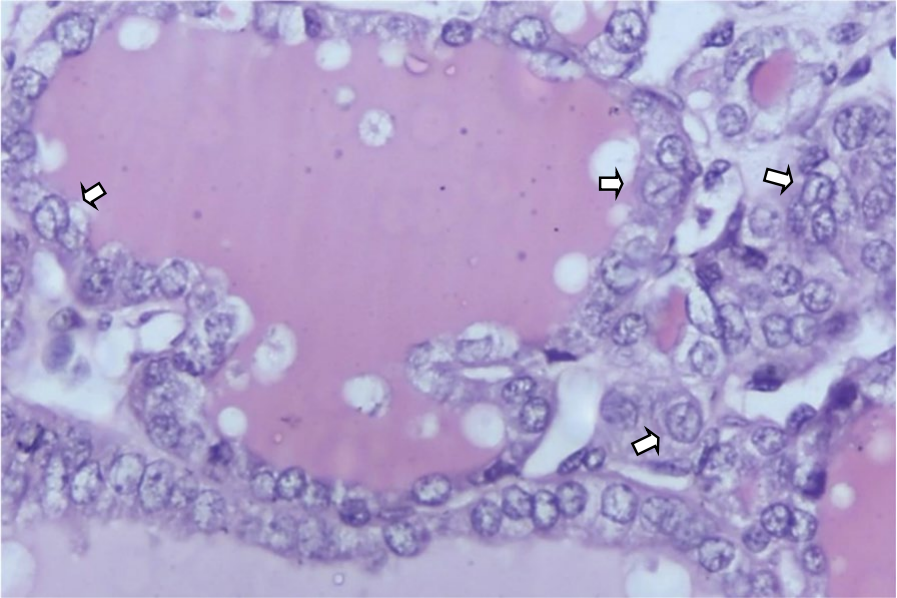
Shows thyroid follicular cells with an oval elongated appearance and longitudinal nuclear groove

The tissue FFPE blocks were of NG, HT, and FA. The hematoxylin and eosin (H&E) stained sections of BTLs were evaluated for the presence of nuclear grooving/high power field (hpf) and a scoring of 0 to 3 was used for the number of grooves. The nuclear grooving was defined as a longitudinal nuclear ridge involving more than half of the nucleus as shown in Fig. 1. Sections with no grooving were scored zero, sections with 1–3 nuclear grooves/hpf, 4–9 nuclear grooves/hpf, and more than 10 nuclear grooves/hpf, were graded 1 + , 2 + , and 3 + respectively. The scoring for nuclear grooving was developed based on the common frequency with which the grooved cells could be identified on H& E sections and areas could be marked for laser capture micro-dissection. The “cut-off” of the number of cells /hpf was defined only for the ease of work with the Laser as one pass of the laser would cut 4–5 adjacent cells, it would help us to know the number of laser passes needed per section and not based on any previous studies or guidelines.

**Fig. 2:**
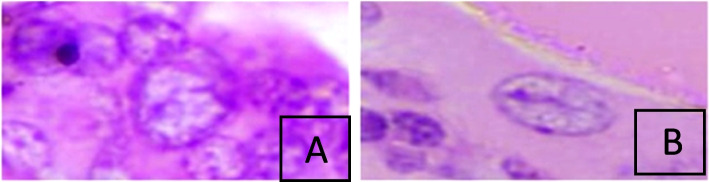
Shows the shape of the thyroid follicular cells with nuclear grooves. **A** Shows a follicular cell with a round nucleus. **B** Shows an oval appearance of the follicular cell

The cells with nuclear grooving were also evaluated for nuclear shape (Fig. 2), the increase in the size of the nucleus, and the presence of nuclear crowding. The size of the nucleus was assessed by comparing it with normal-appearing thyroid tissue, and follicular cells. The nucleus was scored 0 to 1 + with no or minimal nuclear enlargement and was scored 2 + to 3 + if the nucleus was one and a half times to double the size of the normal-appearing follicular cells.

**Fig. 3:**
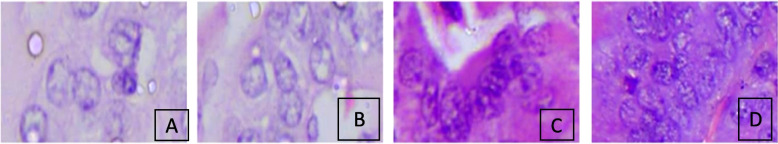
Shows varied nuclear crowding of the follicular cells with nuclear grooving. **A** Shows the crowding of score 0, **B** Score 1 + , **C** Score 2 + , **D** Score 3 + crowding

The nuclear crowding was scored from 0 to 3. The score 0 was no crowding, 1 + minimal, 2 + moderate, and 3 + extensive nuclear crowding as shown in Fig. 3.

### Laser capture microdissection

Multiple sections of 10 μ thickness were cut and deparaffinized. The slides were stained with hematoxylin for the detection of nuclear features. The areas containing cells with nuclear grooving were marked, which were then cherry-picked using Laser-Capture micro-dissection (Palm-Zeiss).

### RT-PCR

About 20 to 50 such cells were micro-dissected in each of the cases and RNA was extracted using a single-cell RNA extraction kit (Arcturus paradise plus reagent, Applied Biosystems) followed by RNA quantification with multiSkan Skyhigh spectrophotometer. The Real-time PCR with Taqman gene expression assay was performed using AgPath-ID™ One-Step RT-PCR kit (Applied Biosystems) using RET/PTC1, RET/PTC3 primer–probe. ACTB gene (TaqMan Gene expression assay, ThermoFischerScientific) was the internal control and PTC samples with known RET/PTC1 and RET/PTC3 translocation were used as a positive control. The primer–probe used in the study is shown in Table 1.

**Table 1:** Shows RET/PTC1, RET/PTC3, and ACTB primer–probe sequences. Rhoden KJ. [13]

RET/PTC1	Forward primer	GAACCGCGACCTGCGCAAA
RET/PTC1	Reverse Primer	CAAGTTCTTCCGAGGGAATTCC
RET/PTC1	Probe	6 FAM- CAA GCG TAA CCA TCG AGG ATC CAA AGT-TAMRA
RET/PTC3	Forward primer	CCCCAGGACTGGCTTACCC
RET/PTC3	Reverse Primer	CAAGTTCTTCCGAGGGAATTCC
RET/PTC3	Probe	6FAM-AAA GCA GAC CTT GGA GAA CAG TCA GGA GG-TAMRA
ACTB	Forward primer	AGC CTC GCC TTT GCC GA
ACTB	Reverse Primer	CTG GTG CCT GGG GCG
ACTB	Probe	VIC- CCG GCT TCG CGG GCG AC—TAMRA

The PCR was performed using 2X RT-PCR Buffer, 12.5 μL, Forward and reverse PCR primers/ TaqMan® probes, 1 μL, 25X RT-PCR Enzyme Mix 1 μL, RNA sample 2–4 μL (based on the RNA quantification) to make up the total volume per reaction to 25 μL. The PCR was set up with conditions as shown in Table 2.

**Table 2:** Shows PCR conditions for the Taqman gene expression assay

Settings	Step 1	Step 2	Step 3	
Stage	Reverse transcription	denaturation	PCR (denaturation)	PCR (anneal/extend)
Temperature	45^0^	95^0^	95^0^	60^0^
time	10 min	10 min	15 s	45 s

**Fig. 4:**
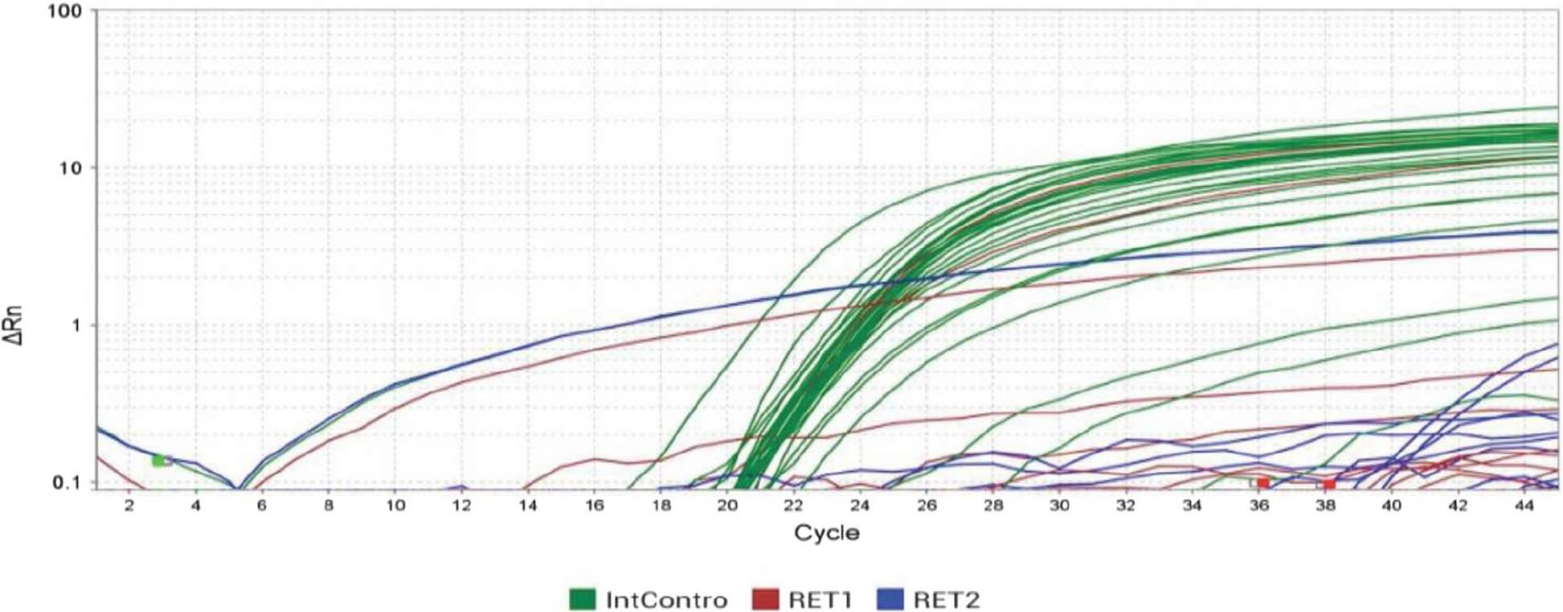
Shows the RT-PCR results in the amplification plot

The RET/PTC translocation results of RT-PCR being a qualitative assay were read as positive or negative as shown in Fig. 4.

### Statistical analysis

The results were compared with various histopathological features for any statistically significant association. Based on the study by Rossella Elisei et al. [14] who showed that 29.2% of the BTLs have RET/PTC rearrangement, on applying the formula n = Z^2^* p * (1-p)/e^2^, where e = error margin of 10%, Z = 1.96 (for 95% confidence limits and α/2 = 0.025), we arrived at a sample size of 83 BTLs samples. We included 87 samples of BTLs diagnosed during the study period.

Statistical tests were done on, www.openepi.com. Descriptive statistics were used was used for the analysis of age and sex distribution. The chi-square test was used for the analysis of various dependent categorical variables and a *p*-value of 0.05 was reported as statistically significant.

## Results

### Histopathological examination

Eighty-seven histopathologically confirmed cases of BTL were included in the study. The male-to-female ratio in the study was 1:5.6. The age group ranges from 20 to 70 yrs with the majority of the cases concerning patients between 31–40 years. Out of 87 BTL cases, 67(77.0%) were NG, 12 (13.7%) were HT, and 8 (9.2%) were FA. The difference in the number of cases in each category reflects the true number of cases seen in our department in each category.

**Fig. 5:**
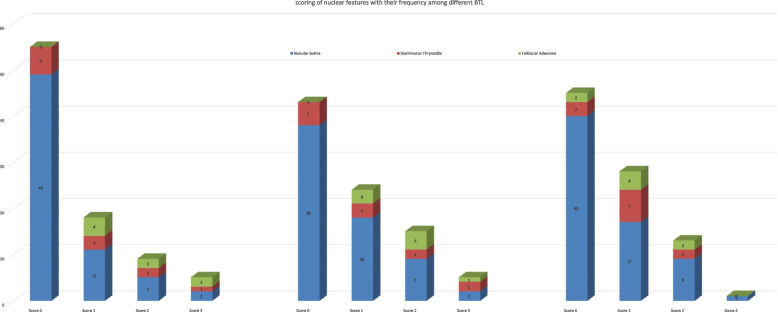
Shows the scoring of nuclear features among different benign thyroid lesions

Out of eighty-seven cases, the number of BTL with different scores of grooving, size, and crowding are described in Fig. 5.

**Fig. 6:**
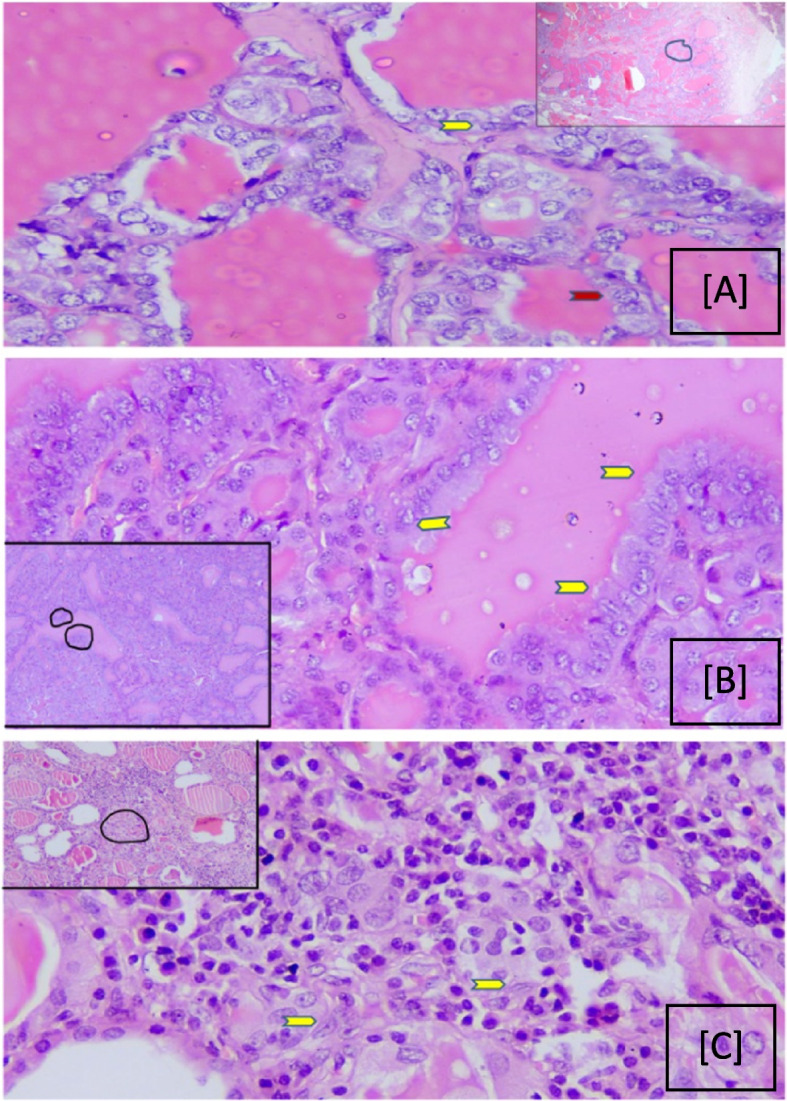
Shows PTC-like nuclear features in various BTLs. **A** Nodular Goiter showing fibrous septae with follicles of varying size in the inset. The area marked in the Inset is been seen in 40 × showing cells with nuclear grooving, round (red arrows, and oval nuclei (yellow arrows). Score 0 to 1 crowding is seen. **B** Follicular adenoma showing follicles of varying sizes. The area marked as in the Inset is been shown in 40 × showing cells with nuclear grooving and oval nuclei (yellow arrowheads). Score 0 to 1 crowding is seen. **C** Hashimoto thyroiditis showing follicles with lymphoplasmacytic infiltrate. The area marked as in the Inset is been shown in 40 × showing cells with nuclear grooving, and oval nuclei (yellow arrowheads), with score 1 crowding and the size of the nucleus increased to score 3

Thirty-two cases (36.8%) had nuclear grooving with 18 out of 67 NG (26.8%), 6 out of 12 HT (50%), and all 8 cases of FA (100%) with a varying number of nuclear grooves as seen in Fig. 6.

### RT-PCR for RET/PTC1 and RET/PTC3 gene translocation

RET/PTC1 and RET/PTC3 translocation were seen in 5 out of 87 (5.74%) cases as seen in Table 3.

**Table 3:** Shows the number of cases showing RET/PTC translocation

	**RET/PTC1**	**RET/PTC3**
NG (Nodular goiter)	0	0
HT (Hashimoto thyroiditis)	2	1
FA (Follicular adenoma)	1^a^	2^a^

^a^One case of FA showed Positivity for both RET/PTC1 and RET/PTC3. No translocation was seen in the nodular goiter

On applying Chi-square Test to look into the association of RET/PTC rearrangement with Histopathological diagnosis, it was seen that a statistically significant association was seen between the histopathological diagnosis and RET/PTC translocation (*p*-value 0.0001) as shown in Table 4.

**Table 4:** Shows an association of histopathological diagnosis with RET/PTC1 and RET/PTC3 translocation

Diagnosis	RET / PTC Translocation		*P* Value
	Positive	Negative	
NG	00 (0.0)	67 (81.7)	0.0001
HT	03 (60.0)	09 (11.0)	
FA	02 (40.0)	06 (07.3)	
Total	05 (100.0)	82 (100.0)	

On assessing the association between various nuclear features with RET/PTC translocation, using the chi-square test it was seen that nuclear grooving with an increase in nuclear size and oval elongated shape had a statistically significant association with RET/PTC translocation as seen in Table 5.

**Table 5:** Shows the association of nuclear features like grooving, size and shape with RET/PTC translocation

Grooving	RET/PTC Translocation		*P* Value
	Positive	Negative	
0	0 (0.0)	55 (67.1)	0.001
1	02 (40.0)	16 (19.5)	
2	01 (20.0)	08 (9.8)	
3	02 (40.0)	03 (3.7)	
total	05 (100.0)	82 (100.0)	
size	RET/PTC Translocation		*P* Value
	Positive	Negative	
0	0 (0.0)	43 (52.4)	0.002
1	01 (20.0)	23 (28.0)	
2	02 (40.0)	13 (15.9)	
3	02 (40.0)	03 (3.7)	
total	05 (100.0)	82 (100.0)	
Shape	RET/PTC Translocation		*P* Value
	Positive	Negative	
Oval	03 (60.0)	12 (14.6)	0.034
Round	02 (40.0)	70 (85.40	
Total	05 (100.0)	82 (100.0)	

On assessing the association between nuclear crowding with RET/PTC translocation using the chi-square test, no statistically significant association between nuclear crowding and RET/PTC translocation was observed (p- 0.097).

On assessing the association between nuclear grooving among different BTL with RET/PTC translocation, using the chi-square test, it was seen that the nuclear grooving in HT was associated with RET/PTC translocation. However, no association between increased grooving with translocation was seen. No statistically significant association between nuclear grooving in FA and NG with RET/PTC translocation was observed (Table 6).

**Table 6:** Shows the association of histopathological diagnosis with nuclear grooving and RET/PTC translocation

Diagnosis	Grooving	Mutation		*P* Value
		Positive	Negative	
FA	1	0 (0.0)	04 (66.7)	0.264
	2	1 (50.0)	01 (16.7)	
	3	1 (50.0)	01 (16.7)	
HT	0	0 (0.0)	06 (66.7)	0.038
	1	02 (66.7)	01 (11.1)	
	2	0 (0.0)	02 (22.2)	
	3	01 (33.3)	0 (0.0)	
NG	0	0 (0.0)	49 (73.1)	1.000
	1	0 (0.0)	11 (16.4)	
	2	0 (0.0)	05 (07.5)	
	3	0 (0.0)	02 (3.0)	

Nuclear grooving showed a statistical association with RET/PTC1 and RET/PTC3 gene rearrangement, using the chi-square test (Table 7).

**Table 7:** Shows the association of nuclear grooving with RET/PTC1 and RET/PTC3 gene translocation

**Grooving**	**RET/PTC 1 Translocation**		***P*** **Value**
	Positive	Negative	
0	0 (0.0)	55 (65.5)	0.0001
1	01 (33.3)	17 (20.2)	
2	0 (0.0)	09 (10.7)	
3	02 (66.7)	03 (3.6)	
Grooving	RET/PTC 3 Translocation		*P* Value
	Positive	Negative	
0	0 (0.0)	55 (65.5)	0.048
1	01 (33.3)	17 (20.2)	
2	01 (33.3)	08 (09.5)	
3	01 (33.3)	04 (04.8)	

## Discussion

The thyroid is a unique endocrine organ where both benign and malignant lesions can co-exist. PTC is the most common thyroid malignancy and is often present along with BTLs like HT or NG more often than, without any pre-existing thyroid pathology [15–17]. Although the histopathological features of PTC have been very well defined, many BTLs do show some nuclear features mimicking PTC. Nuclear grooving is a longitudinal nuclear membrane invagination seen along the long axis of the nucleus of thyroid follicular cells with an oval rather than round appearance. These features are always given significant importance in thyroid cyto-histopathology in differentiating non-neoplastic from neoplastic lesions particularly, in the PTC. But, when nuclear grooves with oval shape, chromatin clearing, and nuclear overlapping, are seen in BTLs, they do cause a diagnostic dilemma, resulting in the diagnosis of “atypical cytology/indeterminate significance” on fine needle aspiration (FNA) samples [3, 18, 19]. C D Scopa et al. studied 80 non-papillary thyroid lesions both neoplastic and non-neoplastic and showed that nuclear grooving was present in a variety of thyroid lesions with 76% of the BTLs having grooves of ≤ 6/hpf [3]. The frequency of nuclear grooving among BTLs in our study was 36.8% but studies have reported the frequency of grooving to range between 3.6% to 52.4% based on the history of irradiation [6, 19]. Various studies have tried to ascertain the significance of nuclear grooving, which are functional channels connecting the nuclear envelope to the chromatin and have been proposed to have a possible role in Ca + + signaling ortransport from the cytoplasm to the nucleus [12, 20]. Some studies have put forth a semi-quantitative approach of counting the number of cells with nuclear grooves and categorizing the lesions as either PTC, indeterminate cytology or benign lesions [21]. Benign thyroid hyperplastic nodules are typically characterized by thyroid follicular cells with small, round, dark nuclei and a honeycombing pattern of arrangement. However, focal nuclear atypia, including grooves, oval shape, chromatin clearing, and overlapping, have been reported in hyperplastic nodules, which leads to diagnostic difficulties and can be mistaken for PTC [18, 19]. Studies have also shown that some of the BTL like FA, HT, and adenomatous goiter show positivity for RET/PTC gene translocation by RT-PTC [7]. Among the BTLs, HT is commonly associated with PTC and micro-papillary tumors [22, 23]. Small clusters of cells do show PTC-like nuclear features, particularly grooving in HT [21, 24, 25]. 

However, there is no definitive demarcation of cells with such PTC-like nuclear features to make a definitive diagnosis of PTC. It could be hypothesized that chronic inflammation with auto-antibody production and T-cell mediated cytotoxic effect in HT, might result in DNA damage and genetic alterations [24]. We observed that 6 out of 12 cases of HT, had grooving, with 3 out of 6 cases being positive for RET/PTC gene translocation. There was a statistically significant association of nuclear grooving in HT with genetic alteration like RET/PTC translocation. Apart from grooving, nuclear enlargement, irregular nuclear membrane, and clearing due to peripheral chromatin condensation were also commonly seen in our study suggesting the possibility of genetic or epigenetic alterations. The study by Dae-Young Kang et al. on normal thyrocytes, oxyphil cells, and PTC cells dissected by Laser capture to study the RET/PTC-RAS-BRAF cascade in these cells showed an increased nuclear expression of RET, RAS, and ERK proteins in oxyphil cells, PTC cells and concluded a molecular link between Hurthle cell metaplasia and PTC progression [26]. 

Our study also favors that genetic alterations like RET/PTC gene translocation results in PTC-like nuclear morphology in HT and that, there could be further additional mutations or alterations in downstream signaling pathways or unknown epigenetic alterations responsible for the overt development of PTC in HT. As studies have shown that PTC associated with HT are usually multifocal and aggressive [22], it is worthwhile looking for the PTC-like nuclear features in HT on cytology, particularly nuclear grooving, as regular follow-up with ultrasound and FNA can be warranted for any increase in the size of the lesion and early detection of PTC. As induction of RET/PTC alterations has shown to induce nuclear irregularities in some studies, genetic testing for RET/PTC alteration in an FNA sample of HT showing increased nuclear grooving with oval and elongated nucleus might aid in the early detection of PTC [27]. Apart from nuclear grooving, many studies have shown intra-nuclear pseudo-inclusions in HT [24]. The intra-nuclear pseudo-inclusions of PTC are distinct and appear as “punched out” areas in the nucleus, whereas in BTLs they appear as vague nuclear clearings.

Follicular neoplasms are another benign lesion showing nuclear grooving. In our study, all 8 cases of FA, showed grooving of varying degrees with RET/PTC gene translocation seen in 2 cases. The follicular lesions range from FA to the newer entities of WHO Classification, the borderline tumors like “Uncertain malignant potential (UMP)” and “noninvasive follicular thyroid neoplasm with papillary-like nuclear features” (NIFTP) [28]. The distinction between FA and the borderline entities is based on the scoring of PTC-like nuclear features, further pressing the significance of nuclear grooves. Although our study did not show any statistical significance between grooving and RET/PTC translocation in FA, closer observation for PTC-like nuclear features will aid in a definitive diagnosis, warranting regular follow-up of the patient for any rapid increase in the size of the lesion and planning further management.

Many non-thyroid lesions and tumors also show nuclear groovings like granulosa cell tumor of the ovary, mesothelioma, and Langerhans cell histiocytosis, to name a few. These lesions have different underlying genetic alterations, pointing to the fact that nuclear irregularities can have multiple causative factors like BRAF mutations in Langerhans cell histiocytosis and FOXL1 gene mutation in ovarian granulosa cell tumor [29].

## Conclusions

To conclude, our study emphasizes, that, nuclear grooving can be seen in BTLs also and is not restricted only to PTCs. The frequency of nuclear grooving among BTLs in our study was 36.8%. Our study shows, that when BTLs, show nuclear grooves, with an increase in the nuclear size, oval and elongated shape, favors the possibility of an underlying genetic aberration like RET/PTC gene translocation, which in turn supports the reporting pathologist to suggest a close follow up of the patients on seeing such nuclear features on cytology or histopathology sample, particularly in HT. However, the limitation of our study was that only RET/PTC 1 and 3 gene translocations were studied and the possibility of other genetic alterations responsible for nuclear irregularities was not looked into. Also, our study did not show, any significant association between the number of nuclear grooves and the rearrangement, making it difficult to assess the “cut-off” level of calling significant nuclear grooving in a clinical setting. To answer our research question of whether the BTLs have RET/PTC gene translocations responsible for nuclear grooving and whether nuclear grooving can be used as a surrogate marker for RET/PTC gene translocation in BTL, although a significant number of HT showed RET/PTC gene translocation, not all grooved cells were positive for the translocation, implying nuclear grooves in histo-cytopathology cannot be used as surrogate markers for RET/PTC gene translocation, but rather occur due to multifactorial causes.

## References

[1] Chan JK, Saw D (1986). The grooved nucleus: a useful diagnostic criterion of papillary carcinoma of the thyroid. Am J Surg Pathol.

[2] Deligiorgi-Politi H (1987). Nuclear crease as a cytodiognostic feature of papillary carcinoma of thyroid in fine needle aspiration biopsies. Diagn Cytopathol.

[3] Scopa CD, Melachrinou M, Saradopoulou C, Merino MJ (1993). The significance of the grooved nucleus in thyroid lesions. Mod Pathol.

[4] Nikiforov YE (2002). RET/PTC Rearrangement in thyroid tumors. Endocr Pathol.

[5] Tallini G, Asa SL (2001). RET oncogene activation in papillary thyroid carcinoma. Adv Anat Pathol.

[6] Romei C, Elisei R (2012). RET/PTC Translocations and Clinico-Pathological Features in Human Papillary Thyroid Carcinoma. Front Endocrinol (Lausanne).

[7] Ishizaka Y, Kobayashi S, Ushijima T, Hirohashi S, Sugimura T, Nagao M (1991). Detection of RET/PTC transcripts in thyroid adenomas and adenomatous goiter by an RT-PCR method. Oncogene.

[8] Wirtschafter A, Schmidt R, Rosen D, Kundu N, Santoro M, Fusco A (1997). Expression of the RET/PTC fusion gene as a marker for papillary carcinoma in Hashimoto’s thyroiditis. Laryngoscope.

[9] Guerra A, Sapio MR, Marotta V, Campanile E, Moretti MI, Deandrea M (2011). Prevalence of RET/PTC rearrangement in benign and malignant thyroid nodules and its clinical application. Endocr J.

[10] Marotta V, Guerra A, Sapio MR, Vitale M (2011). RET/PTC rearrangement in benign and malignant thyroid diseases: a clinical standpoint. Eur J Endocrinol.

[11] Sapio MR, Guerra A, Marotta V, Campanile E, Formisano R, Deandrea M (2011). High growth rate of benign thyroid nodules bearing RET/PTC rearrangements. J Clin Endocrinol Metab..

[12] Fischer AH, Bond JA, Taysavang P, Battles E, Wynford-Thomas D (1998). Papillary thyroid carcinoma oncogene (RET/PTC) alters the nuclear envelope and chromatin structure. Am J Pathol.

[13] Rhoden KJ, Johnson C, Brandao G, Howe JG, Smith BR, Tallini G (2004). Real-time quantitative RT-PCR identifies distinct c-RET, RET/PTC1 and RET/PTC3 expression patterns in papillary thyroid carcinoma. Lab Invest.

[14] Elisei R, Romei C, Vorontsova T, Cosci B, Veremeychik V, Kuchinskaya E (2001). RET/PTC Rearrangements in Thyroid Nodules: Studies in Irradiated and Not Irradiated, Malignant and Benign Thyroid Lesions in Children and Adults. J Clin Endocrinol Metab.

[15] Konturek A, Barczyński M, Wierzchowski W (2013). Coexistence of papillary thyroid cancer with Hashimoto thyroiditis. Langenbecks Arch Surg..

[16] Gandolfi PP, Frisina A, Raffa M, Renda F, Rocchetti O, Ruggeri C (2004). The incidence of thyroid carcinoma in multinodular goiter: retrospective analysis. Acta Biomed.

[17] Nikiforova MN, Caudill CM, Biddinger P, Nikiforov YE (2002). Prevalence of RET/PTC Rearrangements in Hashimoto’s Thyroiditis and Papillary Thyroid Carcinomas. Int J Surg Pathol..

[18] Pusztaszeri MP, Krane JF, Cibas ES, Daniels G, Faquin WC (2014). FNAB of benign thyroid nodules with papillary hyperplasia: a cytological and histological evaluation. Cancer Cytopathol.

[19] Baloch ZW, LiVolsi VA (2014). Current role and value of fine-needle aspiration in nodular goiter. Best Pract Res Clin Endocrinol Metab.

[20] Lui PPY, Lee CY, Tsang D, Kong SK (1998). Ca++ is released from the nuclear tubular structure into the nucleoplasm in C6 glioma cells after stimulation with phorbol ester. FEBS Lett.

[21] Yang YJ, Demirci SS (2003). Evaluating the diagnostic significance of nuclear grooves in thyroid fine needle aspirates with a semiquantitative approach. Acta Cytol.

[22] Hussein O, Abdelwahab K, Hamdy O (2020). Thyroid cancer associated with Hashimoto thyroiditis: similarities and differences in an endemic area. J Egypt Natl Canc Inst.

[23] Prasad ML, Huang Y, Pellegata NS, de la Chapelle A, Kloos RT (2004). Hashimoto's thyroiditis with papillary thyroid carcinoma (PTC)-like nuclear alterations express molecular markers of PTC. Histopathology.

[24] Berho M, Suster S (1995). Clear nuclear changes in Hashimoto’s thyroiditis. A clinicopathologic study of 12 cases. Ann Clin Lab Sci.

[25] Fiore E (2011). Hashimoto’s thyroiditis is associated with papillary thyroid carcinoma: role of TSH and of treatment with L-thyroxine. Endocr Relat Cancer.

[26] Kang DY, Kim KH, Kim JM, Kim SH, Kim JY, Baik HW, Kim YS (2007). High prevalence of RET, RAS, and ERK expression in Hashimoto's thyroiditis and in papillary thyroid carcinoma in the Korean population. Thyroid.

[27] Fischer E (2020). G: Nuclear Morphology and the Biology of Cancer Cells. Acta Cytol.

[28] Kakudo K, Bychkov A, Bai Y, Li Y, Liu Z,  Jung CK (2018). The new 4th edition World Health Organization classification for thyroid tumors, Asian perspectives. Pathol Int.

[29] Batistatou A, Scopa CD. Review Articles: Pathogenesis and Diagnostic Significance of Nuclear Grooves in Thyroid and Other Sites. Int J Surg Pathol. 2009;17(2):107-10. 10.1177/1066896908316071. 10.1177/1066896908316071 18480396

